# Glucocorticoids Affect 24 h Clock Genes Expression in Human Adipose Tissue Explant Cultures

**DOI:** 10.1371/journal.pone.0050435

**Published:** 2012-12-10

**Authors:** Purificación Gómez-Abellán, Antoni Díez-Noguera, Juan A. Madrid, Juan A. Luján, José M. Ordovás, Marta Garaulet

**Affiliations:** 1 Department of Physiology, Faculty of Biology, University of Murcia, Murcia, Spain; 2 Department of Physiology, Faculty of Pharmacy, University of Barcelona, Barcelona, Spain; 3 General Surgery Service, University Hospital “Virgen de la Arrixaca”, Murcia, Spain; 4 Nutrition and Genomics Laboratory, Jean Mayer United States Department of Agriculture Human Nutrition Research Center on Aging, at Tufts University, Boston, Massachusetts, United States of America; 5 Department of Epidemiology, Centro Nacional Investigaciones Cardiovasculares (CNIC), Madrid, Spain; 6 Instituto Madrileño de Estudios Avanzados en Alimentación (IMDEA-FOOD), Madrid, Spain; Karlsruhe Institute of Technology, Germany

## Abstract

**Aims:**

to examine firstly whether *CLOCK* exhibits a circadian expression in human visceral (V) and subcutaneous (S) adipose tissue (AT) *in vitro* as compared with *BMAL1* and *PER2,* and secondly to investigate the possible effect of the glucocorticoid analogue dexamethasone (DEX) on positive and negative clock genes expression.

**Subjects and Methods:**

VAT and SAT biopsies were obtained from morbid obese women (body mass index≥40 kg/m^2^) (n = 6). In order to investigate rhythmic expression pattern of clock genes and the effect of DEX on *CLOCK*, *PER2* and *BMAL1* expression, control AT (without DEX) and AT explants treated with DEX (2 hours) were cultured during 24 h and gene expression was analyzed at the following times: 10:00 h, 14:00 h, 18:00 h, 22:00 h, 02:00 h and 06:00 h, using qRT-PCR.

**Results:**

*CLOCK*, *BMAL1* and *PER2* expression exhibited circadian patterns in both VAT and SAT explants that were adjusted to a typical 24 h sinusoidal curve. *PER2* expression (negative element) was in antiphase with respect to *CLOCK* and in phase with *BMAL1* expression (both positive elements) in the SAT (situation not present in VAT). A marked effect of DEX exposure on both positive and negative clock genes expression patterns was observed. Indeed, DEX treatment modified the rhythmicity pattern towards altered patterns with a period lower than 24 hours in all genes and in both tissues.

**Conclusions:**

24 h patterns in *CLOCK* and *BMAL1* (positive clock elements) and *PER2* (negative element) mRNA levels were observed in human adipose explants. These patterns were altered by dexamethasone exposure.

## Introduction

Circadian rhythmicity is essential to accommodate our physiology to the specific needs of the organism during the 24-hours day cycle. Mammalian circadian rhythms are regulated and synchronized by a master clock located in the hypothalamic suprachiasmatic nuclei (SCN) [1]. In addition, peripheral clocks that maintain circadian oscillations even in the absence of the master clock have been identified in other organs such liver, digestive system or even the adipose tissue (AT) [2], [3].

The first characterized clock component was the transcription factor CLOCK (Circadian Locomotor Output Cycles Kaput) [4] that dimerizes with BMAL1, another component of this system and constitutes the positive limb of the clock [1]. On the other hand, PER2 together with CRY1 constitutes the negative limb in this complex circadian machinery.

CLOCK is the product of the *CLOCK* gene that in both, genetic and experimental studies has been associated with obesity. Indeed, *Clock* mutant mice have been shown to be hyperphagic and obese relative to their wild-type controls [5]. Its deficiency was accompanied by alterations in diurnal rhythms of physical activity, feeding, and metabolic rate [5]. Similarly, in humans, a number of single nucleotide polymorphisms (SNPs) in *Clock* have been correlated with predisposition to obesity [6]–[8], and resistance to weight loss [9].

Previously, we have shown that *PER2* and *BMAL1* together with other genes coding for clock components (i.e., *CRY1)* are expressed in cultured human adipose tissue explants according to a rhythmic pattern [3]. However, it remains to be defined whether *CLOCK* exerts a similar circadian oscillation in human adipose tissue, and if this oscillation differs between visceral and subcutaneous fat depots.

A variety of biochemical factors, including glucocorticoids (GCs), have been shown to have the potential to alter transcription of core clock genes [10]. GCs have potent physiological effects and their levels show marked daily oscillation, which is thought to be driven by the master circadian clock in SCN of the hypothalamus via the hypothalamo-pituitary-adrenal axis [11]. These circadian oscillations are also present in human adipose tissue [3]. GCs bind to glucocorticoid receptors in the cytoplasm of target cells and then the glucocorticoid receptors are transported to the nucleus to act as transcription factors [11]. A relevant question is the potential influence of GCs on clock genes expression. In this sense, although it has been described that GCs are particularly potent at eliciting the rhythmic expression of the mRNAs for peripheral clock genes [11], outcomes remain controversial depending on the tissue and the clock gene studied. In human adipose tissue it has been described that GCs, particularly dexamethasone (DEX) generates circadian gene expression patterns in different clock genes in undifferentiated and adipocyte-differentiated stem cells [12]. In this context, the possible effect of GCs on *CLOCK*, *BMAL1* and *PER2* as representative genes of the positive and negative limb of the peripheral clock genes expression in human adipose tissue cultured remains unknown.

Therefore, the primary aims of this study were to characterize for the first time the rhythmicity of expression of *CLOCK* in human visceral and subcutaneous adipose tissue as compared with *PER2* and *BMAL1*, and whether the glucocorticoid analogue DEX modulated positive and negative clock genes *in vitro*.

## Subjects and Methods

### Subjects

Visceral and subcutaneous abdominal adipose tissue biopsies were obtained from morbid obese non diabetic women (n = 6), aged 44±4 years and body mass index (BMI): 41.2±3.8 Kg/m^2^, undergoing laparoscopic gastric bypass surgery due to obesity at the General Surgery Service of “Virgen de la Arrixaca” Hospital (Murcia, Spain). The women studied were postmenopausal and were not under hormone replacement therapy. The day before surgery, all patients were synchronized having lunch at 14:30 h and dinner at 21:00 h. The adipose tissue biopsies were taken as paired samples from the two adipose tissue depots (visceral and subcutaneous) at the beginning of the surgical procedure (estimated time of biopsies sampling at 13:00 h).

The protocols were approved by the Ethics Committee of the “Virgen de la Arrixaca” University Hospital, and the subjects signed a written informed consent before the biopsies were obtained.

### Clinical characteristics

Arterial pressure, BMI, waist and hip circumference were assessed by standard procedures, while skinfolds (biceps, triceps, suprailiac and subscapular) were measured with a Harpenden caliper (Holtain Ltd, Bryberian, Crymmych, Pembrokeshire, UK). Total body fat (%) was evaluated by bioimpedance with a TANITA TBF-300 (TANITA Corporation of America, Arlington Heights, IL). Sagittal diameter and coronal diameter were measured at the level of the iliac crest (L_4–5_) using a Holtain Kahn Abdominal Cali skinfold. Women were classified in visceral and subcutaneous obesity calculating the index VA/SA (Visceral Area/Subcutaneous Area) after applying the following equation [13]: VA/SApredicted = 0.868+0.064×Sagittal diameter – 0.036×Coronal diameter – 0.022×triceps skinfold. Those patients with VA/SA >0.42 were classified as having visceral obesity [13]. Fasting plasma concentrations of glucose, triacylglycerols, total cholesterol, low-density lipoprotein (LDL) cholesterol and high-density lipoprotein (HDL) cholesterol were determined with standard analytical methods (Roche Diagnostics GmbH, Mannheim, Germany). Basal metabolic Rate (BMR) was calculated from the Harris and Benedict equation [14].

### Adipose Tissue Culture

Immediately after the surgery, explants were placed at 37°C for 24 hour in a humidified atmosphere containing 7% CO_2_ in culture dishes with 12 wells. Approximately 500 mg adipose tissue explants (minimal pieces of 1–2 mm^3^ in order to allow the maximal contact of adipose tissue with the medium) were placed in 2.5 ml of Dulbecco's modified Eagles Medium (DMEM) supplemented with 10% fetal bovine serum (GIBCO) and a mixture of penicillin-streptomycin-glutamine (PSG from GIBCO #10378-016).

On the next day, at 08:00 h a.m., the medium was exchanged with DEX medium (DMEM+PSG, supplemented with 1 µM DEX (0.4 mM in ethanol) (Brunschwig AG, Basel, Switzerland)). In control wells, a control medium 0.25% ethanol in DMEM with PSG was added. After 2 hours, at 10:00 h a.m., these mediums (DEX and control) were replaced with serum-free DMEM+PSG. Then, the adipose explants were collected to perform gene expression analysis at the following times (T): 0, 4, 8, 12, 16 and 20 in which T0 was arbitrarily defined as 10:00 h, T4 as 14:00 h, T8 as 18:00 h, T12 as 22:00 h, T16 as 02:00 h, and T20 as 06:00 h. Gene expression was measured only for one circadian cycle (24 hours), but in the second day of culture. All cultures were performed in duplicate.

### Analysis of Gene Expression

Total RNA was extracted from adipose tissue explants using RNeasy Kit (QIAGEN, Courtabeouf, France). Reverse transcription was performed using random hexamers as primers and Thermoscript® reverse transcriptase (Invitrogen, Cergy Pontoise, France) with 1 µg total RNA for each sample. Quantitative real-time PCR was performed using an ABI PRISM 7000 HT Sequence Detection System (Applied Biosystems, CA, USA), using TaqMan® Universal PCR Master Mix and specific TaqMan probes (Applied Biosystems, CA, USA). The primers used in the study are the following references from Applied Biosystems: *CLOCK* (Hs00231857_m1), *BMAL1* (Hs00154147_m1) and *PER2* (Hs00256143_m1) and *18S* (Hs99999901_s1). *18S* was selected as the housekeeping gene because, as analyzed by repeated measures ANOVA test, no significant differences in circadian rhythmicity in *18S* gene expression were observed in any of the fat depots studied among times (*P*>0.05). Gene mRNA levels were normalized to *18S* using the 2^−ΔΔCt^ method [15].

### Rhythm Calculation and Statistical Analysis

Clinical and anthropometric data are presented as means ± SD. The results for gene expression, expressed in arbitrary units, are presented as means ± SEM.

To investigate the presence of circadian rhythms, least squares periodic regression [16] was used to fit the data, from each group, to a sinusoidal function. This adjustment allows verifying the presence of a circadian rhythm through the significance of fitting and the percentage of variance explained by the rhythm. It also provides the estimation of the characteristic parameters of the rhythm: the amplitude of oscillation, its acrophase (the time the variable reaches the maximum) and the central value (mesor). These parameters are represented graphically in a polar plot (hourly) to visualize the characteristics of the rhythm by a vector (cosinor) whose length corresponds to the amplitude and the direction to the acrophase.

All the data from all individuals are used simultaneously to directly estimate the population parameters (one-step method) using a model that includes a sinusoid and a constant for each individual, which corresponds to the individual mesor, eliminating the effect of interindividual variation in the estimated global rhythm. Confidence intervals of estimated parameters are narrower than in the two-steps method and the estimation of these parameters is more reliable.

The percentages of variance explained by the rhythm are referred to the “corrected variance”, after removing the effect of the mesor. Since in the dexamethasone treated group, the presence of a circadian rhythm was not detected, to investigate shape changes on the daily pattern, we used (in addition to T = 24 h) a sinusoidal function with T = 12 h. This curve is simply a bimodal pattern that produces a depression in the profile of the curve. This pattern fits remarkably well to the profile observed in the experimental results. Thus the sinusoid, with T = 12 h is a good mathematical model to fit the experimental points, without necessarily be interpreted as the presence of a rhythm of 12 h. In this case, the analysis and calculations follow the same steps as in the case of 24 hours, but changing the period. All calculations were performed with an “ad hoc” computer application (“Ritme v4”, @ Antoni Diez-Noguera, Univ. Barcelona, 2012) written in C++ and tested with examples from literature.

Mean circadian gene expression of the total population into each fat depot was analyzed by using repeated measures ANOVA test, with a *post hoc* test of least significant difference (LSD) correction and while differences in PR (Percent Rhythm: percentage of variability accounted for by sinusoidal curve) among control and DEX treated adipose explants from *CLOCK* and *PER2* was analyzed by Wilcoxon non-parametric test. Statistical analysis (ANOVA, means, comparisons, etc) was carried out using the package SPSS for Windows (release 15.0; SPSS Inc, Chicago, US). The level of significance for all statistical tests and hypotheses was set at *P*<0.05.

## Results

### Characteristics of the Population

The women studied were morbidly obese (BMI>40 Kg/m^2^), non diabetic and they had MetS (Table S1 contains baseline characteristics of women studied), according to the International Diabetes Federation (IDF) criteria [17]. VA/SA lower than 0.4 indicates that women did not suffer from visceral obesity. The individual and average values for waist circumference, triglycerides, HDL-cholesterol and systolic pressure exceeded the cut off points proposed by IDF (Table S1).

### Circadian Clock Gene Expression

**Figure 1:**
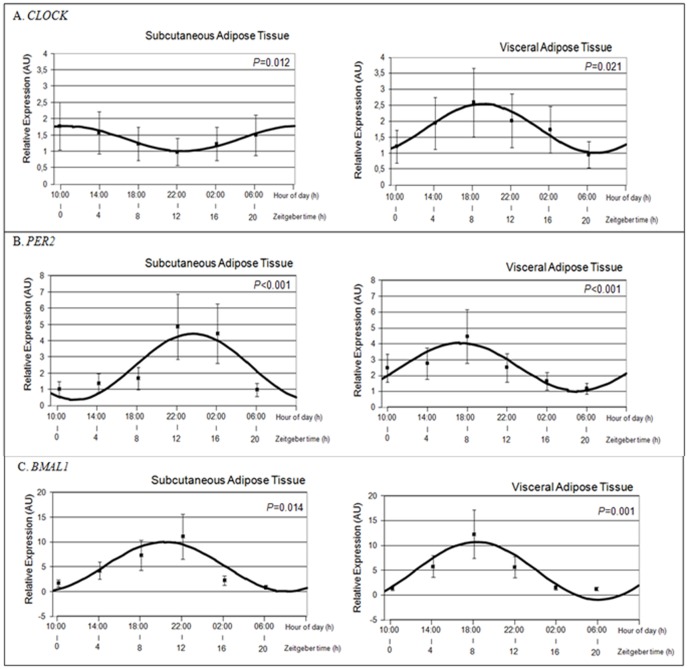
Rhythmic expression of *CLOCK* (1A), *PER2* (1B) and *BMAL1* (1C) in human subcutaneous (S) and visceral (V) adipose tissue (AT) (Control samples). Adipose depots were isolated at 4-h intervals over the course of the day from adipose tissue cultures (time at ZT0 (1000 h), ZT4 (1400 h), ZT8 (1800 h), ZT12 (2200 h), ZT16 (0200 h) and ZT20 hours (0600 h)). Raw data (not mesor corrected) are represented by six black dots. Solid lines represent the estimated 24 h sinusoidal curve, in the population. *CLOCK*, *PER2* and *BMAL1* expression among different times of culture and statistical differences were analyzed in both depots (VAT and SAT) (repeated measures ANOVA test *P*<0.05) evidences changes in gene expression due to time. Data of relative expression are represented as Arbitrary Units (AU). Data are reported as means ± S.E.M.

Data consistently revealed the presence of a circadian rhythm in *CLOCK*, *BMAL1* and *PER2* gene expression in both subcutaneous and visceral adipose tissue. In both depots the presence of rhythm was highly significant (*P*<0.001) (**Table 1**). **Figure 1** graphically represents the circadian pattern of the clock genes studied in both subcutaneous and visceral adipose tissue depots. When relative gene expression among different 24 hours was analyzed by using repeated measures ANOVA test, statistical differences were found in subcutaneous (*CLOCK*: *P* = 0.012; *PER2*: *P*<0.001; *BMAL1*: *P* = 0.014) and in visceral ATs (*CLOCK*: *P* = 0.021; *PER2*: *P*<0.001; *BMAL1*: *P* = 0.001), evidencing timing differences in *CLOCK*, *BMAL1* and *PER2* expression.

**Table 1:** Parameters imputed from periodic regression analysis for genes studied.

1A	*CLOCK*									
	Control Subcutaneous Adipose Tissue					Control Visceral Adipose Tissue				
Population (T = 24 h)	Mesor (AU)	Amplitude (AU)	Acrophase (hh∶mm)	Percent Rhythm (%)	*P*	Mesor (AU)	Amplitude (AU)	Acrophase (hh∶mm)	Percent Rhythm (%)	*P*
Estimated	1.40	0.36	00:12	75.54	**<0.001**	1.78	0.73	08:55	76.00	**<0.001**
Lower limit	1.27	0.27	23:13			1.48	0.55	07:57		
Upper limit	1.53	0.45	01:10			2.08	0.91	09:54		

	Dexamethasone Subcutaneous Adipose Tissue					Dexamethasone Visceral Adipose Tissue				
Population (T = 24 h)	Mesor (AU)	Amplitude (AU)	Acrophase (hh∶mm)	Percent Rhythm (%)	*P*	Mesor (AU)	Amplitude (AU)	Acrophase (hh∶mm)	Percent Rhythm (%)	*P*
Estimated	1.62	0.28	10:43	6.73	0.317	2.51	0.38	21:23	4.66	0.455
Lower limit	1.51	−0.19				2.14	−0.38			
Upper limit	1.73	0.75				2.88	1.14			

Population (T = 12 h)	Mesor (AU)	Amplitude (AU)	Acrophase (hh∶mm)	Percent Rhythm (%)	*P*	Mesor (AU)	Amplitude (AU)	Acrophase (hh∶mm)	Percent Rhythm (%)	P
Estimated	-	1.00	11:57	85.97	**<0.001**	-	1.37	02:40	61.06	**<0.001**
Lower limit	-	0.82	11:37			-	0.88	01:59		
Upper limit	-	1.18	12:18			-	1.86	03:22		

1B	*PER2*									
	Control Subcutaneous Adipose Tissue					Control Visceral Adipose Tissue				
Population (T = 24 h)	Mesor (AU)	Amplitude (AU)	Acrophase (hh∶mm)	Percent Rhythm (%)	*P*	Mesor (AU)	Amplitude (AU)	Acrophase (hh∶mm)	Percent Rhythm (%)	*P*
Estimated	2.41	2.00	13:18	59.99	**<0.001**	2.53	1.52	07:05	73.62	**<0.001**
Lower limit	1.66	1.27	11:53			2.33	1.12	06:03		
Upper limit	3.16	2.73	14:43			2.73	1.93	08:07		

	Dexamethasone Subcutaneous Adipose Tissue					Dexamethasone Visceral Adipose Tissue				
Population (T = 24 h)	Mesor (AU)	Amplitude (AU)	Acrophase (hh∶mm)	Percent Rhythm (%)	*P*	Mesor (AU)	Amplitude (AU)	Acrophase (hh∶mm)	Percent Rhythm (%)	*P*
Estimated	3.88	2.67	05:09	47.87	**<0.001**	8.25	3.32	-	13.2	0.097
Lower limit	3.28	1.43	03:18			6.83	0.48	-		
Upper limit	4.47	3.91	07:00			9.68	7.11	-		

Population (T = 12 h)	Mesor (AU)	Amplitude (AU)	Acrophase (hh∶mm)	Percent Rhythm (%)	*P*	Mesor (AU)	Amplitude (AU)	Acrophase (hh∶mm)	Percent Rhythm (%)	*P*
Estimated	-	1.84	09:26	22.78	**0.014**	-	8.13	00:33	79.43	**<0.001**
Lower limit	-	0.33	07:36			-	6.29	00:07		
Upper limit	-	3.35	11:16			-	9.98	01:00		

1C	*BMAL1*									
	Control Subcutaneous Adipose Tissue					Control Visceral Adipose Tissue				
Population (T = 24 h)	Mesor (AU)	Amplitude (AU)	Acrophase (hh∶mm)	Percent Rhythm (%)	*P*	Mesor (AU)	Amplitude (AU)	Acrophase (hh∶mm)	Percent Rhythm (%)	*P*
Estimated	5.04	4.98	09:51	65.53	**<0.001**	4.87	5.82	07:58	58.21	**<0.001**
Lower limit	3.27	3.37	08:36			1.98	3.62	06:30		
Upper limit	6.82	6.60	11:06			7.75	8.02	09:27		

	Dexamethasone Subcutaneous Adipose Tissue					Dexamethasone Visceral Adipose Tissue				
Population (T = 24 h)	Mesor (AU)	Amplitude (AU)	Acrophase (hh∶mm)	Percent Rhythm (%)	*P*	Mesor (AU)	Amplitude (AU)	Acrophase (hh∶mm)	Percent Rhythm (%)	*P*
Estimated	2.74	1.16	08:43	27.06	**0.005**	2.77	0.71	18:21	7.82	0.2608
Lower limit	2.05	0.31	05:35			2.20	0.38	06:41		
Upper limit	3.43	2.00	11:52			3.35	1.8	06:18		

Population (T = 12 h)	Mesor (AU)	Amplitude (AU)	Acrophase (hh∶mm)	Percent Rhythm (%)	*P*	Mesor (AU)	Amplitude (AU)	Acrophase (hh∶mm)	Percent Rhythm (%)	*P*
Estimated	-	1.65	11:40	55.08	**<0.001**	-	1.72	02:48	46.12	**<0.001**
Lower limit	-	0.99	10:52			-	0.89	01:49		
Upper limit	-	2.31	12:27			-	2.55	03:45		

Estimates of the parameters that characterize the rhythm (amplitude, acrophase and mesor) calculated for population, including the significance level and the percentage of variance explained by the rhythm of *CLOCK* (1A), *PER2* (1B) and *BMAL1* (1C) for control and dexamethasone subcutaneous and visceral human adipose tissue. Upper and lower limits (95% of confidence) are also shown. Mesor population estimates were obtained directly from the individual mesor. Population estimates for Dexamethasone groups are for a 12 h rhythm (circadian rhythm is not present). Acrophase data are referred to ZT. A negative value in the limits indicates the absence of statistically significant rhythm. AU: Arbitrary units, T: time, h: hours, m: minutes.

**Figure 2:**
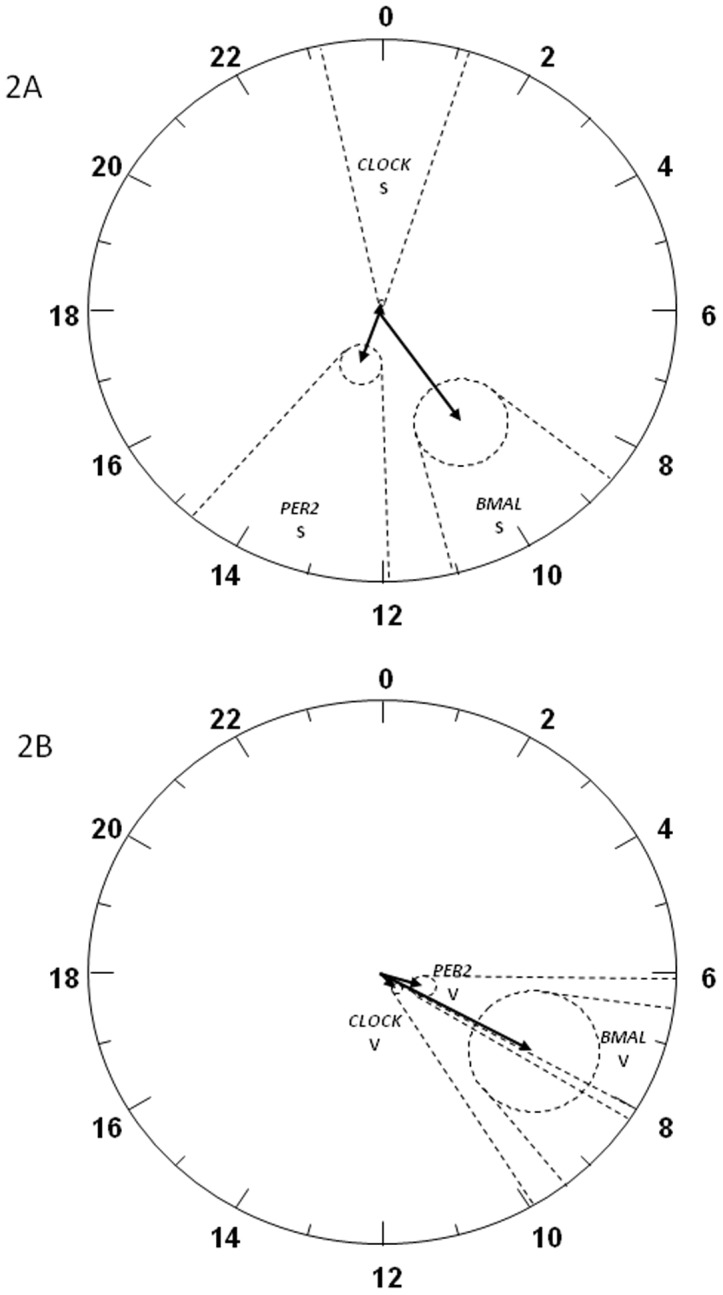
Polar (clock-like) representation of the estimates of the parameters of the rhythm for *CLOCK, PER2* and *BMAL1* in subcutaneous (2A) and visceral (2B) adipose tissue. In the graphs the circles represent 24 hours and the radius corresponds to 10 units. The vector length is the amplitude of the rhythm and it points to its acrophase. Dotted circles are the join 95% confidence limits for the vectors (if limits include the centre, the rhythm is not statistically significant), and dotted lines are the corresponding confidence interval for the acrophase. The population rhythms in control subcutaneous (S) and visceral (V) are clearly visible and statistically significant in both genes studied.

In the graphs (**Figure 2**) the circles represent 24 hours and the radius corresponds to 10 units. The vector length is the amplitude of the rhythm and it points to its acrophase. In **Figure 2** two relevant observations are noted, a) The presence of rhythmicity is clearly visible for all clock genes studied in both AT locations (2A: subcutaneous AT and 2B: visceral AT) taking into account that the ellipses of confidence did not include the center of graph and b) more importantly, *PER2* expression (negative element) was in antiphase with respect to *CLOCK* (positive element) but in phase with *BMAL1* (other representative gene of the positive limb of the clock) in the subcutaneous adipose tissue (Figure 2A). A situation that was not present in visceral adipose tissue where the acrophases of all studied genes were similar. Concretely *PER2* acrophase in visceral AT was only 2 hours in advance of the acrophase of *CLOCK* and 1 hour in advance with respect to *BMAL1* acrophase (Figure 2B).

### Effect of dexamethasone exposure on circadian clock genes expression pattern

**Figure 3:**
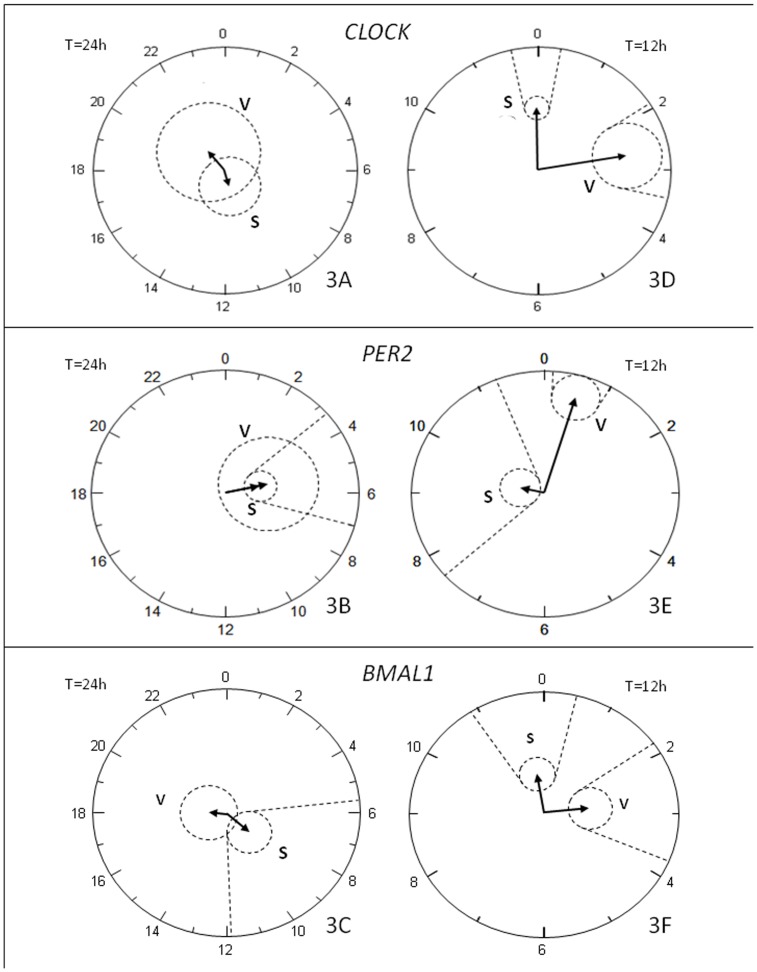
Polar (clock-like) representation of the estimates of the parameters of the rhythm for *CLOCK, PER2* and *BMAL1* in dexamethasone treated groups. In graphs 3A, 3B and 3C the circles represent 24 hours while in 3D, 3E and 3F it represents 12 hours, and the radius corresponds to 5 units. The vector length is the amplitude of the rhythm and it points to its acrophase. Dotted circles are the join 95% confidence limits for the vectors (if limits include the centre, the rhythm is not statistically significant), and dotted lines are the corresponding confidence interval for the acrophase. In 3A, 3B and 3C the absence of 24 hours rhythmicity is evident except for the subcutaneous *PER2* and *BMAL1* that is statistically significant. In 3D, 3E and 3F the presence of an approximately 12 hours oscillation is evidenced in all treated groups.

We observed a marked effect of dexamethasone (DEX) exposure (2 hours) on *CLOCK*, *BMAL1* and *PER2* circadian expression pattern with absence of circadian rhythms in both depots studied after DEX treatment for *CLOCK* (*P*>0.05) (Table 1) while for *BMAL1* and *PER2* this effect was only present for visceral AT (Table 1). The absence of rhythmicity in subcutaneous and visceral fat for *CLOCK* and in visceral AT for *BMAL1* and *PER2* after DEX treatment was also clearly visible in the polar representation (T = 24) in which the ellipses of confidence included the center of graph (**Figure 3**).

**Figure 4:**
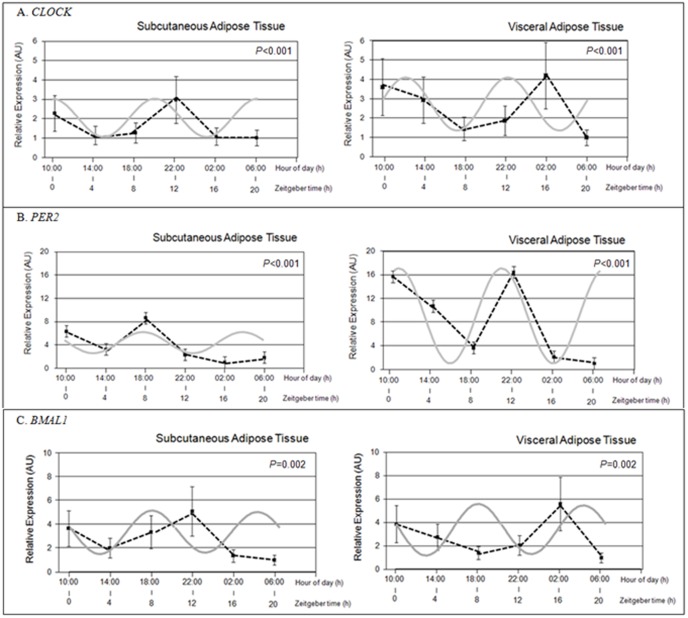
Effect of acute dexamethasone exposure on circadian expression of *CLOCK* (1A), *PER2* (1B) and *BMAL1* (1C) in human subcutaneous (S) and visceral (V) adipose tissue (DEX samples). Adipose depots were isolated at 4-h intervals over the course of the day from adipose tissue cultures (time at ZT0 (1000 h), ZT4 (1400 h), ZT8 (1800 h), ZT12 (2200 h), ZT16 (0200 h) and ZT20 hours (0600 h)). Raw data (not mesor corrected) are represented by six black dots. Solid and gray lines represent the fitted 12 h sinusoidal curve. Differences on the expression among different times of culture and statistical differences were analyzed in both depots (VAT and SAT) (repeated measures ANOVA test, *P*<0.05) evidences changes in time. Data of relative expression are represented as Arbitrary Units (AU). Data are reported as means ± S.E.M.

If using a sinusoid with a period of 12 hours for the population analysis (**Figure 4**), we observed that the clock genes expression rhythms were different when treated with DEX as compared with control (**Figure 1**). One important aspect is that for all the clock genes studied there was a change in the patterns of gene expression that was reflected in an adjustment to a sinusoidal curve of approximately 12 hours after DEX treatment. Indeed, we observed, in subcutaneous depot, immediately after the treatment with DEX that the expression of *CLOCK* decreased at ZT 4 (14:00 h) while it experienced a further increase after 8 hours reaching a peak at ZT 12 (22:00 h). A similar situation happened in visceral adipose tissue, with a significant decrease at ZT8 (18:00 h) and reaching its maximum at ZT 16 (02:00 h) (Figure 4). Similar results were observed for *BMAL1* and *PER2* (Figure 4). This change in the gene expression pattern is probably a result of the direct action of the dexamethasone that would produce a decrease in the expression of clock genes studied in the range of about 6 hours after the administration.

As in control samples, when we analyzed the relative gene expression among different times using repeated measures ANOVA test in DEX treated samples, statistical differences were found evidencing the rhythmicity of clock genes expression after treatment.

## Discussion

The first objective of the present research was to characterize for the first time in human visceral and subcutaneous adipose tissue the rhythmicity of expression of *CLOCK* as compared with *BMAL1* and *PER2*. In agreement with our hypothesis, we have demonstrated in humans the circadian rhythmicity of the *CLOCK* expression in both subcutaneous and visceral adipose tissue in culture conditions. This is consistent with previous observations for *BMAL1*, *PER2* and other clock genes (*CRY1*) [3], cortisol-related genes [18] and some adipokines [19], [20] using similar experimental approaches. Moreover, in the present study the significant *P-values* obtained by periodic regression analysis in both adipose tissue depots show that *CLOCK*, *BMAL1* and *PER2* expression adjusts to a typical 24 h sinusoidal curve (see Table 1 and Figure 1). This fact is also clearly visible in the polar representation in which the ellipses of confidence did not include the center of graph (Figure 2).

Another relevant observation is the phase delay shown between the different adipose tissue locations for *CLOCK*. In fact, we observed that *CLOCK* in both adipose tissue depots, subcutaneous and visceral, oscillated in anti-phase. Thus acrophase in visceral adipose tissue was significantly delayed 8 h with respect to subcutaneous depot suggesting that the internal circadian regulation acts differently in both adipose tissues. Similar expression patterns have been observed for some glucocorticoids (GCs) metabolism-regulating genes, such as glucocorticoid receptor (*GR*) and 11β-hydroxysteroid dehydrogenase type 1 (*11βHSD1*) [18]. Although the molecular mechanisms underlying the distinct regulation of both peripheral adipose tissue depots by GCs remain currently largely unknown, our results are consistent with the notion that the effects of GCs on the gene networks that regulate many key functions in human adipose tissue differ between subcutaneous and visceral fats [21]. Moreover, adipose tissue glucocorticoid action relies on local enzymatic interconversion between *11βHSD1* and *11βHSD2* and glucocorticoid receptor (GR) availability, and both situations differ between subcutaneous and visceral fat [22]. On the other hand, corticoids secreted by the adrenal cortex could activate not only GR but also the mineralocorticoid receptor (MR) both of which are found in adipocytes. This activation could affect *CLOCK* circadian expression. Indeed, Tanaka *et al.* have demonstrated that aldosterone induces circadian gene expression of clock genes in H9c2 cardiomyoblasts and this mechanism is mediated through MR [23].

An interesting result found in the current work is that *PER2* expression (negative element) was in antiphase with respect to *CLOCK* (positive element) in the subcutaneous adipose tissue. These data are in accordance with previous human studies performed confined to less invasive measurements in peripheral blood, oral mucosa and skin [24]–[26], and with studies of adipose tissue biopsies performed in both humans [27], [28] and animal models [29], showing an *in vivo* similar pattern of clock gene expression, exemplified by the anti-phase oscillation of negative and positive mRNAs. However in the current work we observed that *BMAL1* (positive element) oscillated with approximately the same phase as *PER2* (negative element). This unusual phase relationship has also been described in our previous study performed *in vitro* in human adipose tissue in culture conditions [3], and in other *in vivo* work in human peripheral blood mononuclear cells [30] and in mouse bone marrow [31]. In this sense it is important to remember that in addition to *PER2*, other genes are involved in the regulation of *BMAL1* in the molecular circadian oscillator. Indeed, *BMAL1* expression is under control of the “secondary clock loop” genes such as *RORs*, *REV-ERBs* and *PPARα*, which are linked to regulation of metabolism as well. Therefore, further investigations should determine if any change in the expression of these other components could be influencing the timing of *BMAL1* expression. It could be also interesting to measure differences between (*in vitro* and *in vivo*) studies in a similar set of obese patients.

Differently to what happened in the subcutaneous adipose tissue, in the visceral region the acrophases of three genes studied were similar. Concretely *PER2* acrophase was only 2 hours in advance of the acrophase of *CLOCK* and 1 hour in advance as compared to *BMAL1* acrophase (Figure 2B). These findings are in agreement with a previous study where subcutaneous adipose tissue is classified as healthy adipose tissue [32], whereas that in the visceral AT, some disturbances occur in clock genes expression that could be related to the pathophysiology of this adipose region and its implications on the metabolic syndrome.

**The second objective** of our work was to investigate the possible effect of the glucocorticoid analogue DEX on both positive and negative clock genes expression in human adipose tissue *in vitro*. In this regard, our data revealed a marked effect of DEX exposure (2 hours) on *CLOCK*, *BMAL1* and *PER2* circadian expression patterns with absence of circadian rhythms in both depots studied after DEX treatment for *CLOCK* while for *BMAL1* and *PER2* this effect was only present for visceral fat. However, if using a sinusoid with a period of 12 hours for the population analysis, highly significant adjustments (*P*<0.01) were obtained in the two depots for the three genes studied. These altered patterns characterized by a loss of a typical 24 h sinusoidal curve adjustment is probably a result of the direct action of the dexamethasone that would produce a decrease in the expression of clock genes in the range of about 6 hours after the administration, which gives the plot a different aspect that would explain the adjustment to a sinusoidal curve of approximately 12 hours. It should be noted that the absence of the rhythm of 24 hours in visceral AT for the three genes studied and in subcutaneous AT only for *CLOCK*, may be due to the superposition of the effect produced by administration of DEX. Moreover, the time of administration of DEX could be relevant and action could probably depend on whether administration was performed close to the higher expression (achrophase) of the clock genes analyzed.

Of note, the overall effect of DEX seems to be to make the diurnal pattern in both adipose tissues temporally equivalent. Even so, when we analyzed clock gene expression among different times of culture using repeated measures ANOVA test, statistical differences were found in both depots treated with DEX, evidencing changes in *CLOCK, BMAL1* and *PER2* expression over the time. From these results we could hypothesize that GCs are particularly potent altering the circadian expression of both positive and negative clock genes in human adipose tissue. Interestingly, our current data in human adipose tissue are consistent with previous findings of Balsalobre *et al.*, who have shown that a DEX shock transiently changes the phase of circadian gene expression in peripheral tissues of experimental animals [33]. Moreover, we have replicated this behavior which is consistent with the DEX induced *CLOCK* expression pattern previously described in bovine neutrophils [34]. The rhythmic secretion of GCs and their ability to phase shift peripheral clocks in adipose tissue, makes them valid candidates for signal establishing the link between the SCN pacemaker and the peripheral oscillators in adipose tissue [10].

One limitation of the current work was that all of the samples were from morbidly obese women. We must be cautious to extrapolate data to obese or normal weight subjects. In fact, the lack of a non-obese control group makes interpretation of the results more difficult, also regarding potential differences in glucocorticoid status between obese and non-obese individuals. However, to obtain enough AT sample from normal-weight individuals may be challenging and it could be also an ethical problem, considering the difficulties in obtaining enough fat from visceral and subcutaneous locations within the same patients to perform twelve different adipose tissue explants cultures (six for control and six for dexamethasone cultures).

In summary, 24 h patterns in *CLOCK* and *BMAL1* (positive clock elements) and *PER2* (negative element) mRNA levels were observed in human visceral and subcutaneous adipose tissue explants and these patterns were altered by an acute activation of glucocorticoid signalling making the diurnal pattern in both adipose tissues temporally equivalent. The time of administration of dexamethasone is important because action will depend on whether this administration is performed close to the higher expression (achrophase) of clock genes, but clearly dexamethasone treatment modified the rhythmicity pattern towards altered patterns with a period lower than 24 hours in all genes and in both tissues.

## Supporting Information

Table S1 General characteristics of the population studied. Data are presented as means ± SD. Bold characters indicate values higher than cut-off points proposed by the International Diabetes Federation (IDF) [17]. BMI: Body Mass Index. WC: Waist circumference. HC: Hip Circumference. WHR: Waist to Hip Ratio. BMR: Basal Metabolic Rate. VA/SA_predicted_: Visceral Area/Subcutaneous Area_predicted_ [13]. HDL: high-density lipoprotein; LDL: low-density lipoprotein.
(DOCX)
