# Supplementary material for: Glucocorticoids Affect 24 h Clock Genes Expression in Human Adipose Tissue Explant Cultures
Source: PLoS One. 2012 Dec 10;7(12):e50435. doi: 10.1371/journal.pone.0050435 (PMC3519463; doi:10.1371/journal.pone.0050435)
Supplement: Table S1 — General characteristics of the population studied. Data are presented as means ± SD. Bold characters indicate values higher than cut-off points proposed by the International Diabetes Federation (IDF) [17]. BMI: Body Mass Index. WC: Waist circumference. HC: Hip Circumference. WHR: Waist to Hip Ratio. BMR: Basal Metabolic Rate. VA/SApredicted: Visceral Area/Subcutaneous Areapredicted [13]. HDL: high-density lipoprotein; LDL: low-density lipoprotein. (DOCX) [file pone.0050435.s001.docx]

**Table S1. General characteristics of the population studied.**

|  | Patients (n=6) |
| --- | --- |
| Age (y) | 44 ± 4 |
| Weight (Kg) | 106.8 ± 11.3 |
| Height (cm) | 160.8 ± 2.3 |
| BMI (Kg/m^2^) | 41.2 ± 3.8 |
| Body fat (%) | 49 ± 2 |
| WC (cm) | **127 ± 11** |
| HC (cm) | 138 ± 8 |
| WHR | 0.92 ± 0.07 |
| Sagittal Diameter (cm) | 33 ± 2 |
| Coronal Diameter (cm) | 49 ± 1 |
| Bicipital skinfold (mm) | 33 ± 6 |
| Tricipital skinfold (mm) | 41 ± 3 |
| Subscapular skinfold (mm) | 44 ± 4 |
| Suprailliac skinfold (mm) | 41 ± 3 |
| BMR (Kcal) | 1772 ± 115 |
| VA/SA_predicted_ | 0.33 ± 0.16 |
| Glucose (mmol/l) | 5.18 ± 0.44 |
| Cholesterol (mmol/l) | 5.43 ± 0.99 |
| Triglycerides (mmol/l) | **1.87 ± 1.24** |
| HDL-cholesterol (mmol/l) | **1.27 ± 0.28** |
| LDL-cholesterol (mmol/l) | 3.91 ± 1.02 |
| Systolic Pressure (mmHg) | **147 ± 22** |
| Diastolic Pressure (mmHg) | 72 ± 12 |

Data are presented as means ± SD. Bold characters indicate values higher than cut-off points proposed by the International Diabetes Federation (IDF) [17]. BMI: Body Mass Index. WC: Waist circumference. HC: Hip Circumference. WHR: Waist to Hip Ratio. BMR: Basal Metabolic Rate. VA/SA_predicted_: Visceral Area/Subcutaneous Area_predicted_ [13]. HDL: high-density lipoprotein; LDL: low-density lipoprotein.
